# Effect of Nanocellulose Types on Microporous Acrylic Acid/Sodium Alginate Super Absorbent Polymers

**DOI:** 10.3390/jfb13040273

**Published:** 2022-12-05

**Authors:** Saeed Ismaeilimoghadam, Mehdi Jonoobi, Yahya Hamzeh, Serena Danti

**Affiliations:** 1 Department of Wood and Paper Science and Technology, Faculty of Natural Resources, University of Tehran, Karaj 77871-31587, Iran; 2 Pisa Research Unit (UdR), Consorzio Interuniversitario Nazionale per la Scienza e Tecnologia dei Materiali (INSTM), 50121 Florence, Italy; 3 Department of Civil and Industrial Engineering, University of Pisa, 56122 Pisa, Italy

**Keywords:** polysaccharides, super absorbent polymers, rheological properties, porosity, nanocellulose

## Abstract

The aim of this study was to investigate the effect of different types of nanocellulose, i.e., cellulose nanocrystal (CNC), cellulose nanofiber (CNF) and bacterial nanocellulose (BNC), and also different drying methods (oven-drying and freeze-drying) on the properties of acrylic acid (AA)/sodium alginate (SA) super absorbent polymers (SAPs). In addition, the presence of ammonium per sulfate as an initiator and *N-N* methylene-bis-acrylamide as a cross-linker were considered. Synthesized SAPs were characterized by Fourier transform infrared (FTIR) spectroscopy and scanning electron microscopy (SEM). The absorption and rheological properties (i.e., storage modulus and loss modulus) were also investigated. The results of FTIR spectroscopy demonstrated several types of interactions, such as hydrogen and esterification, between SA, AA and nanocellulose. SEM analysis revealed a microporous structure in the SAPs. All SAPs had a centrifuge retention capacity (CRC)/free swelling capacity (FSC) ≥ 69%. The absorption behavior showed that the oven-dried SAPs had superior (about 2×) CRC and FRC in different aqueous media compared to the freeze-dried counterparts. The freeze-dried SAPs showed increased rheological properties in comparison to the oven-dried ones, with SAPs containing BNC and CNC having the highest rheological properties, respectively. Overall, it can be concluded that oven-dried SAPs containing CNC had better absorption properties than the other ones tested in this study.

## 1. Introduction

Super absorbent polymers (SAPs) are a class of hydrogels, able to absorb and retain high amount of liquids with respect to their mass, which are formed by the polymerization of one, or a combination of different types of monomers, interacting through physical or chemical interactions. The absorbent ability of SAPs comes from the hydrophilic functional groups of the polymer chains, such as hydroxyl, carboxyl, amide and amine groups, while their stability and insolubility in water arises from their cross-links [1]. A wide range of materials (e.g., monomers and polymers) from synthetic, acrylic acid [2] and acrylamide [3], to natural starch [4], nanocellulose [5], chitosan [6] and alginate [7], are used to prepare SAPs. Hydrogels have attracted the attention of researchers all over the world for many reasons, including their ability to absorb a large amount water and other fluids [8], drug delivery [9,10], biodegradability [11,12] and their ability to be used in agriculture [13].

Among the different monomers used for synthesizing SAPs, acrylic acid (AA) is one of the most attractive compounds due to its low cost and high absorption properties [14]. However, recent research has increasingly been focused on polysaccharide-based SAPs, looking for a set of properties along with good water absorption capacity, namely, non-toxicity, sustainability and biodegradability [15,16]. Sodium alginate (SA), as well as cellulose-based SAPs, are polysaccharide-based SAPs that not only have an acceptable absorption capacity, but also feature acceptable biocompatibility and biodegradability [12,13,14,15,16,17]. SA is a natural water-soluble polymer composed of (1–4) β-D mannuronic acid and α-L guluronic acid units, forming an irregular pattern along the polymer chains. SA is one of the widely available biological materials used in biomedicine and health products due to its unique properties, i.e., gelling properties, the ability to absorb water and its stabilizing property [17]. Cellulose is another polysaccharide, which represents the most abundant bio-polymer on earth. Nanocellulose is a nanometric form of cellulose, which can be found as three different types; cellulose nanocrystal (CNC), cellulose nanofiber (CNF) and bacterial nanocellulose (BNC). CNC and CNF are plant-derived, but BNC is produced with bacteria [18]. Graft-copolymerization is a technique to synthesize SAPs with a good absorption capacity and good biodegradability at the same time. In this technique, monomers, e.g., AA, and a bio-polymer, e.g., SA, are linked together [7].

In recent years, numerous studies have been conducted on semi bio-based SAPs. Tan Luo et al. (2018) synthesized an SAP based on BNC and AA and investigated its absorption capacity. After the fabrication of SAPs, the effect of different factors, including the AA/BNC ratio, the initiator and cross-linker percentage and the neutralization percentage of AA, on water absorption properties were investigated. The results showed that by increasing the AA/BNC ratio up to 6:1, the water absorption capacity was increased and then decreased to the ratio of 8:1. They also discovered optimal concentrations of the initiator, cross-linker and neutralization percentages to maximize the water absorption capacity [5]. Makhado et al. (2020) reported the absorbent properties of hydrogels based on AA and SA, where the absorption capacity of hydrogels improved by 26%, by adding SA to AA [19]. Shahzamani et al. (2020) investigated the water absorption behavior of a hydrogel made of CNF and AA in distilled water and a saline solution (0.9% NaCl). The results showed that the water absorption of the hydrogels increased with the increase in CNF percentage. On the other hand, the absorption capacity in the saline solution was much lower than that in distilled water [20]. Mohd Amin et al. (2012) synthesized hydrogels based on BNC and AA and investigated their water absorption behavior. The gel fraction (GF) of the resulting hydrogels enhanced with the increase in AA compared to BNC. The results of the water absorption tests also showed that with an increase in the BNC/AA ratio, the water absorption capacity increased considerably [21]. Finally, Thakur and Arotiba (2018) studied the absorbent properties of nanocomposite hydrogels based on AA grafted with SA. TiO_2_ nanoparticles and *N-N* methylene-bis-acrylamide were used as inorganic nanoparticles and cross-linker, respectively. The results showed that the effect of TiO_2_ nanoparticles and SA had a positive effect on the absorption capacity of nanocomposite hydrogels and increased their hydrophilicity [22].

Some research on the grafting of different polysaccharides with synthetic monomers, such as AA, has focused on cellulose, starch and SA, among others. However, comprehensive research on the synthesis of three-component SAPs of AA, SA and nanocellulose has not been reported in the literature. Furthermore, there has not been any investigation into a comparison of the different types of nanocellulose and their effects on the functional properties of the resulting SAPs. Therefore, in this research, three different kinds of nanocellulose, namely, CNC, CNF and BNC were used to synthesize SAPs, and their influence on the properties of AA/SA SAP in the presence of APS as an initiator and NMBA as a cross-linker were investigated. Additionally, two drying methods, including oven-drying and freeze-drying were used to obtain dried SAPs. Synthesized SAPs were characterized by Fourier transform infrared (FTIR) spectroscopy and scanning electron microscopy (SEM). The absorption and rheological properties (i.e., storage modulus and loss modulus) were also studied. Manufacturing highly performing SAPs with an increased content of natural polymers, such as cellulose, would improve the eco-sustainability and biocompatibility of SAP-containing products. Such SAPs could find application in hygienic and personal care products, i.e., baby diapers, sanitary napkins and adult incontinence pads. Having SAPs with a higher absorption capacity than that of the commercial SAPs (i.e., 60 g/g), would allow industries to use less powders in their pads, thus increasing performance, reducing volume and weight and improving their waste management procedures.

## 2. Materials and Methods

### 2.1. Materials

AA with a purity of 99% and a molecular weight of 72.08 g/mol as a monomer, APS with a molecular weight of 218.19 g/mol as an initiator, NMBA with a purity of 99% and a molecular weight of 154.17 g/mol as a cross-linker, medium viscosity SA with a molecular weight of 225.6 g/mol, sodium chloride (NaCl) and sodium hydroxide (NaOH) were all obtained from Sigma-Aldrich (St. Louis, MO, USA). Different types of nanocellulose, including CNC, CNF and BNC were obtained from Nano Novin Polymer Company (Tehran, Iran). The characteristics of these nanomaterials are shown in Table 1.

### 2.2. Preparation of Different Types of Nanocellulose

CNC was purchased as a white powder and no preparation was required. However, CNF was purchased as a gel (suspension) with dry content of 2%. Application of CNF at this concentration greatly reduced the solid percentage of SAPs. Therefore, the water content of CNF suspension was reduced by a centrifuge at 3500 rpm until the dry content of the suspension increased up to 5% and then it was used for the production of SAPs. BNC was purchased as a hydrogel pad (pellicle). First, the BNC pellicles were completely disintegrated by a laboratory homogenizer for 30 min at 10,000 rpm. Then, to reduce water content, it was centrifuged at 3500 rpm to reach a concentration of about 5%, and then it was used for the manufacturing of SAPs.

### 2.3. SAPs Preparation

**Figure 1:**
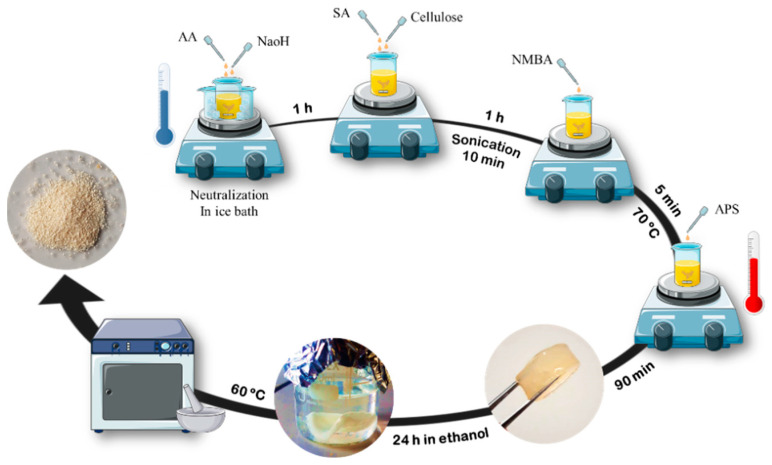
Schematic image of the SAPs’ manufacturing method.

For the preparation of SAPs, the AA/SA ratio was considered 70:30. The nanocellulose percentage was 2 w% based on the weight of AA and SA. It is worth noting that for the addition of nanocellulose, the same amount of SA was reduced, meaning that nanocellulose replaced SA. The neutralization degree of AA was considered to be 10% for all formulations. The initiator and cross-linker were used at 6% and 0.4% based on the weight of the monomer, respectively. The solid percentage of hydrogels was also 20%. First, a specific amount of AA (62.8% by the total weight of materials) was neutralized (10% of AA weight) by a 25 w/v% solution NaOH (6.28% by the total weight of materials) in an ice bath for 1 h with continuous stirring (i.e., with a magnetic stirrer at 500 rpm). In parallel, a specific amount of SA (25.13% by the total weight of materials) was previously prepared as an 8 w/v% solution in distilled water with magnetic stirring for 24 h. The prepared SA along with a certain amount of different nanocellulose (1.8% by the total weight of materials) were added to the neutralized AA. Stirring continued for another 1 h outside the ice bath (200 rpm) until a completely homogeneous suspension was obtained. Then, the obtained homogeneous suspension was subjected to ultrasonic treatment (Bransonic cleaner, Thermo Fisher Scientific Inc., Waltham, MA, USA) for 10 min under a frequency of 100 Hz cycles consisting of 7 s treatment and 3 s rest. After that, a specific amount of cross-linker (0.25% by the total weight of materials) was added to the abovementioned suspension in the form of a 2 w/v% solution in distilled water, and the stirring continued for 5 min at the same stirring speed. In the next step, a specific amount (3.77% by the total weight of materials) of 4 w/v% APS solution in distilled water was added to the obtained suspension, as a reaction initiator. At the same time, to reach the required solid percentage (20%), water was added to the mixture. The temperature of the solution increased with an average heating rate of 5 °C/min and after reaching the desired temperature (70 °C), the temperature remained constant. As the temperature of the solution increased, its viscosity decreased and the stirring speed was gradually increased until it reached 1000 rpm. Stirring continued until a gel was formed. Then the stirring was stopped, and the hydrogel was kept in a hot water bath until the reaction completed (90 min). After 90 min, the hydrogel was removed from the hot bath and cooled at room ambience. Then, the produced gel was cut into small pieces and was soaked in ethanol for 24 h, in order to remove the impurities and also to dehydrate the hydrogel. The hydrogels were transferred to an oven with a temperature of 60 °C afterwards. The oven-dried SAPs were crushed by a mortar and reached the standard size by a laboratory sieve (passed from sieve # 30 and remained on sieve # 50). For the freeze-dried samples, the produced gels were placed in distilled water for 24 h and then placed in a freezer at −20°C for 5 h. Finally, the frozen samples were transferred to the freeze dryer (VirTis AdVantage wizard 2.0 SP Scientific) and frozen at −40 °C and dried for 24 h. After that, the samples were crushed by a mortar and sieved to the standard size. The schematic image of the SAPs’ manufacturing method is shown in Figure 1.

### 2.4. Fourier Transform Infrared Spectroscopy (FTIR)

FTIR spectra was used to investigate the presence and intensity of hydrophilic functional groups in the manufactured SAPs, and also to investigate the possible interactions created in the SAPs. For this purpose, a NICOLET T380 model device (Thermo Fisher Scientific Inc.) was used.

### 2.5. Scanning Electron Microscopy (SEM)

For microscopic photography, first, some powders were completely dried in the oven and then they were completely covered by a thin layer of gold. An electron microscope model of Ion Company, Hillsboro, OR, USA was used for microscopic photography.

### 2.6. Free Swelling Capacity (FSC)

To perform this test, first, 0.1 g (dry weight) of the synthesized SAPs was placed in 40 mL of saline solution (0.9% NaCl) for 30 min and 24 h, separately. After the mentioned times, the swollen gel was filtered by filter paper for 10 min to remove excess water from the swollen hydrogel. The swollen gel was weighed again, and the water absorption capacity was calculated according to Equation (1):

(1) $$\mathrm{FSC}=\frac{m_{t}-m_{o}}{m_{o}}$$

where FSC is the free swelling capacity (g/g), m_t_ is the weight of the swollen sample at the time t (g), and m_o_ is the weight of the completely dry powder (g).

To measure the free swelling capacity in distilled water for 30 min and 24 h, this was performed according to the abovementioned method and relationship, the only difference was that the samples were immersed in 100 mL of distilled water instead of 40 mL of saline solution.

### 2.7. Centrifuge Retention Capacity (CRC)

To conduct this test, according to the absorption capacity test, 0.1 g of completely dry powder was immersed in saline solution (0.9% NaCl) for 30 min and then filtered through filter paper for 10 min. Then, the swollen gel was transferred into a polypropylene fabric (non-woven) and was completely fastened by metal clips, so that the gel could not escape from the fabric. The fabric containing the swollen gel was transferred into the centrifuge falcons, so that they were not in contact with the falcon ends, and the samples were centrifuged for 3 min at 1400 rpm. Then, the falcons were removed from the machine, and the centrifuged gel was weighed again. The CRC was calculated according to Equation (2):

(2) $$\mathrm{CRC}=\frac{m_{c}-m_{o}}{m_{o}}$$

where CRC is the centrifuge retention capacity (g/g), m_c_ is the weight of the gel after centrifugation (g), and m_o_ is the dry weight of the sample before immersion (g).

### 2.8. Gel Fraction (GF)

In order to perform this test, first, 0.1 g of each sample was immersed in 100 mL of distilled water for 24 h. After 12 h, the water was changed and at the end of 24 h, the swollen gel was filtered by filter paper for 10 min and then transferred to the oven with a temperature of 60 °C. GF was calculated according to Equation (3):

(3) $$\mathrm{GF}=\frac{m_{a}}{m_{o}}\times 100$$

where GF is gel fraction (%), m_a_ is the dry weight of the sample after immersion (g), and m_o_ is the dry weight of the sample before immersion (g).

### 2.9. Absorption Under Load (AUL)

**Figure 2:**
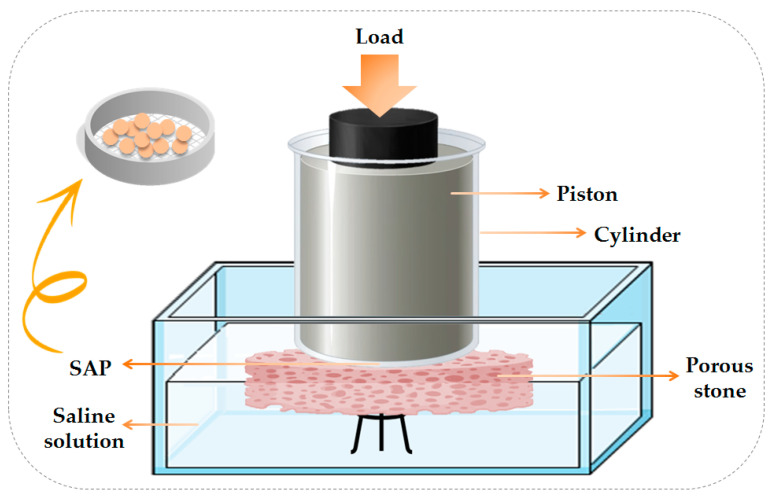
Schematic design of Absorption Under Load (AUL) equipment used to perform experiments of SAPs containing different types of nanocellulose.

To measure this test which simulates the time when the child is sitting and urinating at the same time, special measuring equipment was needed. Figure 2 shows the schematic design of this equipment. First, 0.5 g of completely dry powder was spread uniformly on the metal mesh placed in the cylindrical chamber of the device. Then, a special piston was placed on the powder and the piston assembly, SAPs and the container containing the metal mesh were placed on a special porous stone. A weight was then placed on the piston, so that it could create a pressure of 0.3 pounds per square inch (PSI) on the powder. Then, saline solution (0.9% NaCl) was poured into the device area until the lower half of the thickness of the porous stone was inside the water and the upper half of its thickness was outside the water (Figure 2). In this case, the SAPs had no contact with water and could only absorb it from the porous stone under a pressure of 0.3 PSI. With the increase in the absorption and swelling of the powder, the distance between the piston and the powder increased, and the SAPs had a higher absorption capacity. After 30 min, the absorption process was stopped, the swollen gel was weighed again and the absorption under load was calculated by the Equation (1).

### 2.10. Salt Sensitivity Index (SSI)

The salt sensitivity index is the ratio of the 30 min absorption capacity of saline solution to the 30 min absorption capacity of distilled water, and it indicates the sensitivity of the hydrogel to sodium ions (Na+). The lower the number of this index, the more sensitive the hydrogel is to sodium ions and vice versa.

### 2.11. Ratios

The ratios of CRC/FSC and AUL/FSC, 24 h/30 min absorption capacity in saline solution and distilled water, were calculated according to Equation (4), Equation (5) and Equation (6), respectively.

(4) $$\mathrm{CRC}/\mathrm{FSC}=\frac{\mathrm{CRC}}{\mathrm{FSC}}\times 100$$

(5) $$\mathrm{AUL}/\mathrm{FSC}=\frac{\mathrm{AUL}}{\mathrm{FSC}}\times 100$$

(6) $$\mathrm{FSC}\left(24\;h\right)/\mathrm{FSC}\left(30\;\min\right)=\frac{\mathrm{FSC}24}{\mathrm{FSC}30}\times 100$$

### 2.12. Water Diffusion Coefficient (WDC)

From a theoretical point of view, the water diffusion coefficient is calculated by the slope of the water absorption curve and using Equation (7), where M_t_ is the water absorption at time t, M_sat_ is the saturated point (in this case, 30 min absorption capacity), K and *n* are constant coefficients. To calculate the water diffusion coefficient, it must first be determined that the water absorption behavior of the SAPs is in accordance with Fick’s theory. For this purpose, we can use Equation (8), which is the logarithmic form of Equation (7). When the graph of the logarithm of time is drawn against the logarithm of the water absorption at time t to the maximum water absorption ratio (M_t_/M_sat_), the X coefficient obtained from its regression equation will be the value of (*n*). Such a value lower than 0.45 indicates that the water absorption behavior of the SAPs follows Fick’s theory. After making sure that the water absorption behavior of the SAPs follows Fick’s law, the water diffusion coefficient can be calculated using Equation (9), where L is the thickness of the sample (m) and D is the water diffusion coefficient (m^2^/s). By this equation and considering the slope of the linear part of the M_t_/M_sat_ curve against t^0/5^/L, we can draw the water diffusion coefficient diagram. After drawing the graph, the square of the coefficient X of the regression equation is multiplied by the π number and divided by 16 to calculate the water diffusion coefficient. In order to calculate the water diffusion coefficient, the SAPs were immersed in saline solution for 30 min and sampling was conducted at time intervals of 30 s, 1 min, 2 min, 5 min, 15 min and 30 min. Their water absorption capacity was then calculated and used to calculate the water diffusion coefficient, as shown below:

(7) $$\frac{M_{t}}{M_{\mathrm{sat}}}=Kt^{n}$$

(8) $$\mathrm{LOG}\left\{\frac{M_{t}}{M_{\mathrm{sat}}}\right\}=\mathrm{LOG}\left(K\right)+\mathrm{nLOG}\;\left(t\right)$$

(9) $$\frac{M_{t}}{M_{sat}}=\frac{4}{L}{\left\{\frac{D}{\pi }\right\}}^{0.5}t^{0.5}$$

### 2.13. Rheological Properties

To measure the rheological properties, including storage modulus (G′) and loss modulus (G″), firstly 0.05 g of the synthesized powders was immersed in a saline solution for 30 min, and then the swollen gel was filtered for 10 min to remove excess water. Then the swollen gel was transferred to the rheometer model of Anton Paar, Germany, for rheological examination. All the measurements were performed at 27 °C (laboratory temperature), the gap between the parallel plates was 1 mm and the strain rate was 1%. The frequency investigated to measure storage modulus and loss modulus was in the range of 0.1–100 Hz.

### 2.14. Statistical Analysis

The statistical analysis was performed using the Statistical Package for Social Science (SPSS software, Version 16; IBM SPSS Statistics, Armonk, NY, USA). Univariate technique was used for analyzing the data, and the Duncan test was used for grouping and comparing the averages at a 95% confidence level. Five samples were used for each treatment (*n* = 5).

## 3. Results

### 3.1. FTIR Analysis

**Figure 3:**
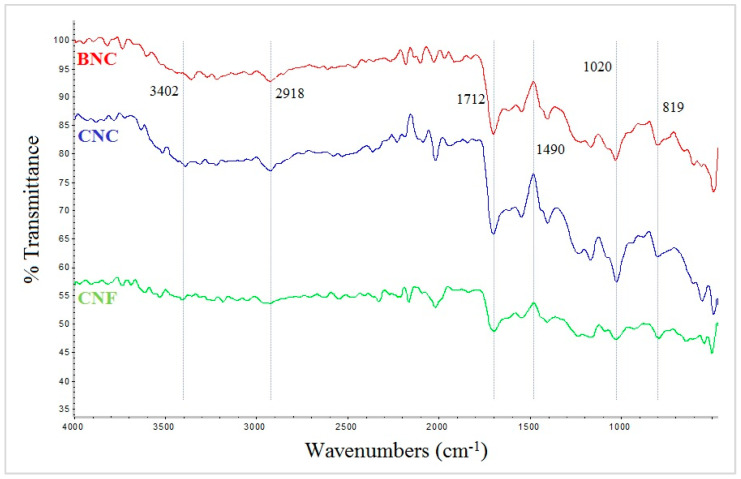
FTIR spectra of SAPs with different types of nanocellulose: BNC, CNC and CNF.

The comparative investigation of the infrared spectra of SAPs containing different types of nanocellulose is shown in Figure 3. According to this Figure, the peak at 3402 cm^−1^ is related to the stretching vibration of hydroxyl groups (O-H). The peak at 2918 cm^−1^ is related to C-H stretching vibration. The intense peak related to the stretching vibration of carbonyl groups (C=O) of carboxylic acid in acrylic acid can be seen at 1712 cm^−1^. The peak corresponding to 1490 cm^−1^ is related to the symmetric stretching vibration of carboxylate groups (COO^−^). 

The peak at 1020 cm^−1^ is related to C-O stretching vibration. This peak represents the structure of polysaccharides and confirms the presence of guluronic and mannuronic acid units of SA. The peak at 819 cm^−1^ is related to the Na-O bond in SA.

### 3.2. SEM Analysis

**Figure 4:**
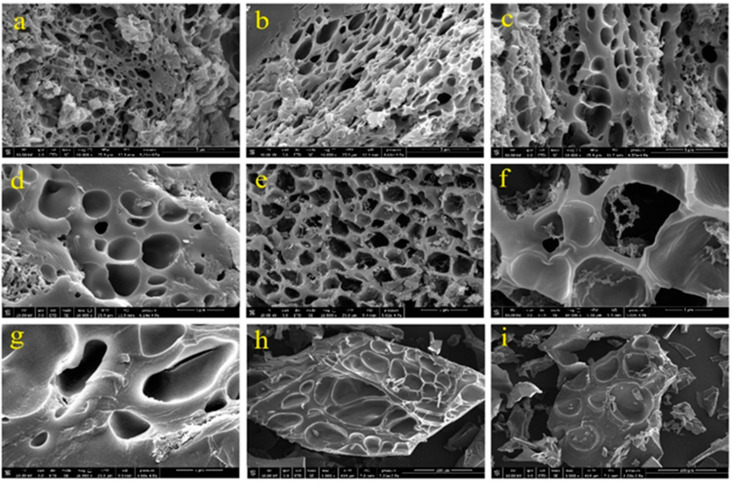
SEM images of super absorbents, super absorbents containing CNC and oven-dried (**a**,**b**), CNF and oven-dried (**c**,**d**), BNC and oven-dried (**e**,**f**), CNC and freeze-dried (**g**), CNF and freeze-dried (**h**), BNC and freeze-dried (**i**).

Figure 4 shows the comparison of SEM micrographs related to the SAPs containing different types of nanocellulose and diverse drying methods. As shown in the SEM micrographs, in the oven-dried SAPs, the distribution of pores had a regular morphology, regardless of the type of nanocellulose. This indicated an appropriate distribution of nanoparticles in the hydrogel network, which occurred as a consequence of ultrasonic treatment.

In the images related to the SAPs containing CNC (Figure 4a,b), the size of the pores is slightly smaller than those in the SAPs containing CNF (Figure 4c,d) and BNC (Figure 4e,f). In the SEM micrographs related to SAPs containing BNC, the presence of these nanofibers at the entrance of the holes was quite clear, which could strongly affect the absorption properties of the SAPs (Section 3.3). On the other hand, in the freeze-dried SAPs the shape of the holes was uneven. The size of the holes was very large in some places, which made the size of the holes bigger than the size of the particles (Figure 4g–i). In some areas, the presence of ice crystals was clearly visible.

### 3.3. Free Swelling Capacity (FSC)

**Figure 5:**
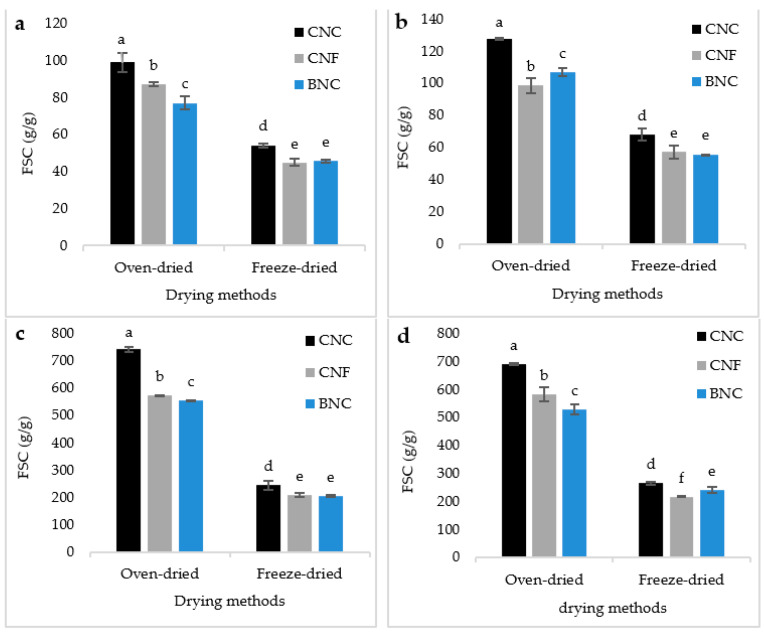
FSC in a saline solution for 30 min (**a**), in a saline solution for 24 h (**b**), in distilled water for 30 min (**c**) and in distilled water for 24 h (**d**). There is a significant difference at the 95% confidence level between the different treatments. Duncan’s grouping is also shown on the graph bars. On the top of the bars, the letter “a” represents the best treatment, followed by “b”, “c”, and so on, in alphabetical order.

The outcomes of the free swelling capacity of SAPs in saline solution for 30 min and 24 h are reported in Figure 5a,b. As demonstrated, the maximum FSC in the saline solution was related to the oven-dried SAPs containing CNC. On the other hand, the minimum FSC in the saline solution was related to the freeze-dried SAPs containing CNF and BNC. The same trend was observed for the FSC in 24 h. However, the FSC of oven-dried SAPs containing BNC was higher than that of the oven-dried SAPs containing CNF. In 30 min, the trend of the FSC was the opposite. It is worth mentioning that the oven-dried SAPs had a higher FSC compared with the freeze-dried ones. In addition, the SAPs containing CNC had a higher absorption capacity than that containing CNF and BNC. The free swelling capacity of SAPs in distilled water for 30 min and 24 h are reported in Figure 5c,d. It could be observed that the FSC in distilled water was higher than that in the saline solution.

### 3.4. Centrifuge Retention Capacity (CRC) and Gel Fraction (GF)

**Figure 6:**
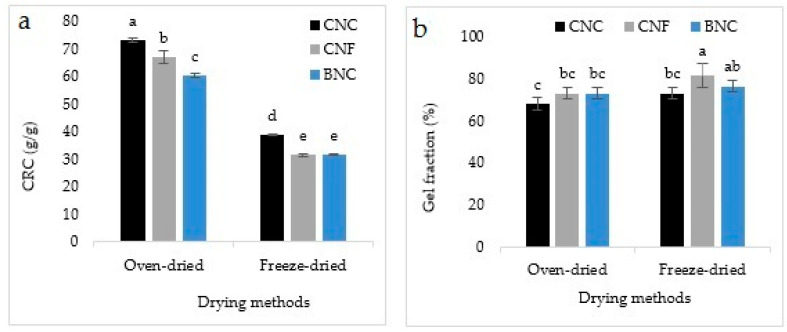
Centrifuge retention capacity (CRC) (**a**) and Gel Fraction (GF) (**b**) of SAPs. There is a significant difference at the 95% confidence level between the different treatments. Duncan’s grouping is also shown on the graph bars. On the top of the bars, the letter “a” represents the best treatment, followed by “b”, “c”, and so on, in alphabetical order.

The CRC and GF of SAPs are represented in Figure 6a,b. According to Figure 6a, between the SAPs containing different types of nanocellulose, the CRC was higher in those containing CNC. Meanwhile, the CRC was higher in oven-dried SAPs compared to the freeze-dried ones. In Figure 6b, the maximum and minimum GF percentage was found in the freeze-dried SAPs containing CNF and the oven-dried SAPs containing CNC, respectively. 

### 3.5. Absorption Under Load (AUL) and Salt Sensitivity Index (SSI)

**Figure 7:**
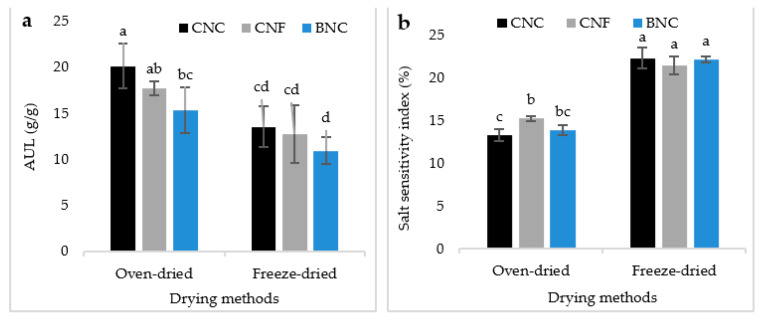
Absorption Under Load (AUL) (**a**) and Salt Sensitivity Index (SSI) (**b**) of SAPs. There is a significant difference at the 95% confidence level between the different treatments. Duncan’s grouping is also shown on the graph bars. On the top of the bars, the letter “a” represents the best treatment, followed by “b”, “c”, and so on, in alphabetical order.

Figure 7 shows the AUL and SSI of the synthesized SAPs. AUL was higher in the oven-dried SAPs compared to the freeze-dried ones and in SAPs containing CNC compared to the other types of nanocellulose (Figure 7a). HoweverSSI was higher in the freeze-dried SAPs. SAP containing CNF exhibited a higher SSI than the other SAPs.

### 3.6. Ratios

**Figure 8:**
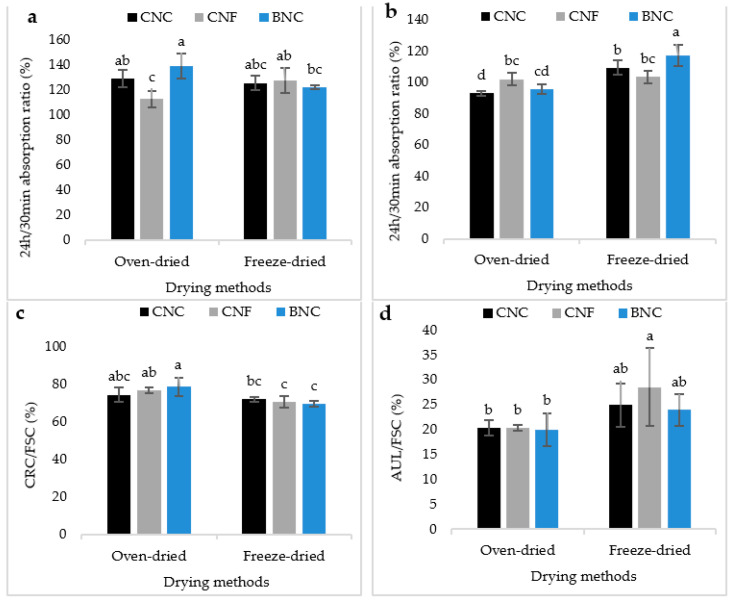
The 24 h/30 min FSC ratio in the saline solution (**a**), in distilled water (**b**), CRC/FSC ratio (**c**) and AUL/FSC ratio (**d**). There is a significant difference at the 95% confidence level between the different treatments. Duncan’s grouping is also shown on the graph bars. On the top of the bars, the letter “a” represents the best treatment, followed by “b”, “c”, and so on, in alphabetical order.

The 24 h/30 min FSC ratio in the saline solution and in distilled water, CRC/FSC ratio and AUL/FSC ratio are displayed in Figure 8. The maximum 24 h/30 min FSC ratio in the saline solution was found in the oven-dried SAPs containing BNC and the minimum was found in the oven-dried SAPs containing CNF (Figure 8a). In any event, in distilled water, this ratio was at a maximum in the freeze-dried SAPs containing BNC and it was at a minimum in the oven-dried SAPs containing CNC (Figure 8b). As shown in Figure 8c, the CRC/FSC ratio is at a maximum in the oven-dried SAPs containing BNC and in the freeze-dried SAPs containing BNC and CNF. Figure 8d shows the AUL/FSC ratio of SAPs. Even though AUL was higher in the oven-dried SAPs than the freeze-dried ones, AUL/FSC ratio was higher in the freeze-dried SAPs than the oven-dried ones.

### 3.7. Water Diffusion Coefficient

**Figure 9:**
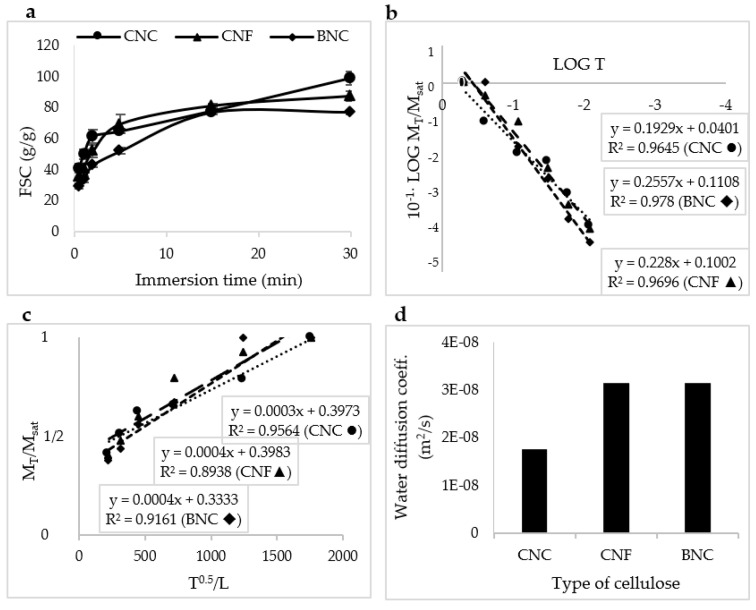
The effect of different types of nanocellulose on the water absorption behavior, and absorption behavior at different times (**a**), LOG T vs. LOG M_T_/M_sat_ (**b**), the calculation method of water diffusion coefficient (**c**), and a bar graph showing the water diffusion coefficients (**d**), in which “E” is for exponential, i.e., 10×.

The effects of the type of nanocellulose on the water absorption behavior and the water diffusion coefficient of the manufactured SAPs is shown in Figure 9. In the first seconds of immersion up to about 120 s, the SAPs containing CNC absorbed a higher amount of water, while the absorption capacity of SAPs containing BNC was the lowest (Figure 9a). This is consistent with the different absorption capacity of these types of SAPs. The water diffusion coefficient model was used to find out the water absorption rate of the manufactured SAPs. As seen in Figure 9b, the *x* coefficient (or *n* value) for SAPs containing CNC, CNF, and BNC was 0.19, 0.22 and 0.25, respectively. Therefore, the water absorption behavior of the manufactured SAPs was in accordance with Fick’s law. Figure 9c was used to calculate the water diffusion coefficient. Since the slope of the curve for SAPs containing CNF and BNC was steeper than that for SAPs containing CNC, the water absorption rate was higher in these types of SAPs. This was confirmed by the value of the water diffusion coefficient (Figure 9d).

**Figure 10:**
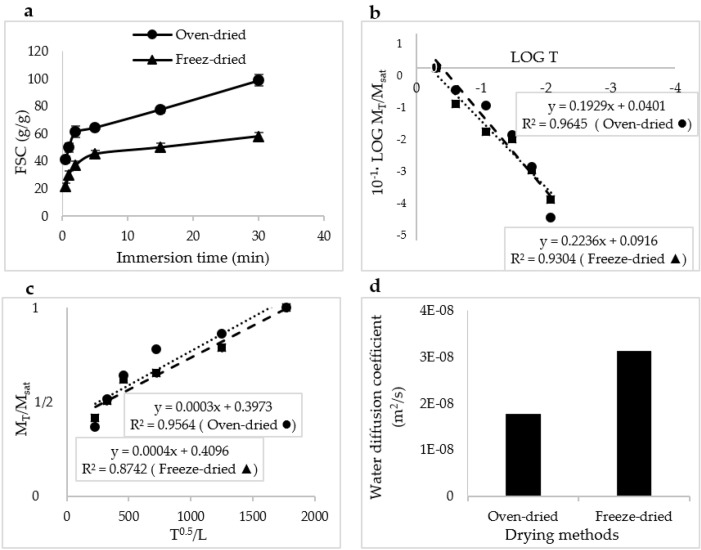
The effect of drying methods on the water absorption behavior, and absorption behavior at different times (**a**), LOG T vs LOG M_T_/M_sat_ (**b**), the calculation method of the water diffusion coefficient (**c**), and a bar graph showing the water diffusion coefficients (**d**), in which “E” is for exponential, i.e., 10×.

The effect of the drying methods on the water absorption behavior and water diffusion coefficient of the manufactured SAPs is reported in Figure 10. As shown in Figure 10a, the water absorption capacity of the oven-dried SAPs was much higher than that of the freeze-dried SAPs. The coefficient *x* (or the *n* value) for the oven-dried and freeze-dried SAPs, were equal to 0.19 and 0.22, respectively (Figure 10b). The slope of the curve for the freeze-dried SAPs was slightly steeper than this slope in the oven-dried SAPs (Figure 10c). This demonstrated the high speed of water absorption in this type of SAP and was confirmed by the value of the water diffusion coefficient (Figure 10d).

### 3.8. Rheological Properties

**Figure 11:**
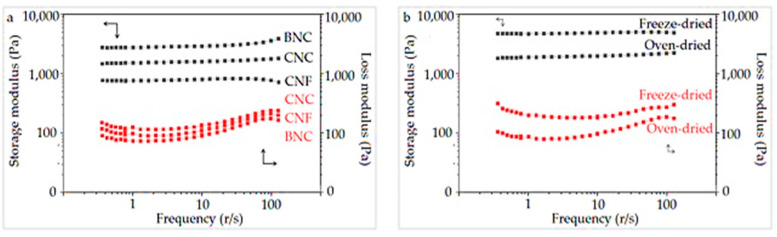
Effect of SAPs with different types of nanocellulose (**a**) and effect of drying methods (**b**), on the rheological properties of SAPs. Arrows represent respective axes related to storage modulus and loss modulus.

Figure 11a displays the effect of the type of nanocellulose on the rheological properties of the manufactured SAPs. The highest storage modulus (G′) was detected in SAPs containing BNC and the lowest in SAPs containing with CNF. Even though the loss moduli (G″) of SAPs were similar, the highest and lowest loss moduli were found in the SAPs containing CNC and BNC, respectively.

A noteworthy point was the large difference between the storage and loss modulus in these types of SAPs, where the loss modulus was much lower than the storage modulus. Figure 11b shows the effects of the drying method of the SAPs over their rheological properties. The storage and the loss modulus of the freeze-dried SAPs were higher than those obtained when the oven-dried procedure was applied.

All the reported results about absorption properties are summarized in Table 2, which clarifies that the oven-dried SAPs containing CNC showed, in most cases, the best properties compared to the other SAPs.

## 4. Discussion

Due to waste management and eco-sustainable material emergence in large consumption products, such as hygiene products, researchers and industries are greatly interested in developing SAPs with a higher content of bio-based polymers, as well as providing high performances for these products, thus opening routes for replacing, as much as possible, fossil-based with bio-based materials.

In this research, the effects of different types of nanocellulose and different drying methods on the properties of AA/SA SAPs were investigated. To conduct a comprehensive evaluation of the produced materials, several analyses were performed, including FTIR, SEM, absorption tests and rheological characterization. FTIR spectroscopy revealed that the intensity of the peaks related to carboxylic acid groups [23,24,25,26] and hydroxyl groups [27,28] in SAPs containing CNC was higher than in SAPs containing BNC and CNF. The intensity of the peak at 1490 cm^−1^, related to the symmetric stretching vibration of carboxylate groups (COO^-^) [19,20,21,22,23,24,25,26,27,28,29], was also lower in SAPs containing CNF compared to the other two. Due to the presence of water and temperature (70 °C) in the process of synthesizing SAPs, the possibility of forming an ester bond (esterification reaction) between the carboxyl groups of AA and the hydroxyl groups of nanocellulose is high [30]. Due to having many amorphous areas in its structure, CNF has a better reactivity with AA compared to CNC and BNC. The low intensity of the peaks related to the carboxyl and hydroxyl groups in the SAPs containing CNF was due to the greater ester reaction between the hydroxyl groups of CNF and the carboxyl groups of AA. It is worth mentioning that, besides the presence of ester bonds, there is also the possibility of hydrogen bonds between the hydroxyl groups of SA and nanocellulose. In this case, the chance of establishing hydrogen bonds between the hydroxyl groups of SA with hydroxyl groups of CNF is greater than that of CNC and BNC.

SEM analysis highlighted the presence of pores. In the process of synthesizing SAPs, the presence of water plays a very important role in their properties, both chemically and physically. In fact, after the formation of the gel and upon drying of the SAPs, a large quantity of pores was created when the water was evaporated. It is expected that the water escaped mainly from the weak points of the hydrogel, such as the location of nanoparticles. Therefore, the presence of CNC, CNF and BNC can influence the morphology of SAPs as these nanoparticles could create more pores in the SAPs. As mentioned earlier, BNC fibers are located in the entrance of holes in the SAPs. It may seem that these fibers are aggregated, but since these fibers have a very high aspect ratio of about 350 [31], it is very likely that the curl index of these fibers is very high, which increases the interweaving of these nanofibers.

Based on the results of FSC in a saline solution, it is evident that with the increase in the crystallinity of nanocellulose, their reactivity also decreased. Among the nanomaterials, CNC has a crystallinity of about 85–90% [32], more than the crystallinity of CNF (65–75%) [33] and even BNC (75–85%) [34]. This was also confirmed by the FTIR analysis, where it was found that the number of ester bonds formed between the carboxyl groups of AA and the hydroxyl groups of CNF was higher than the formation of the abovementioned bonds in SAPs containing CNC and BNC. Therefore, in SAPs containing CNC, more hydrophilic carboxyl groups in AA are free and had the ability to absorb water. For this reason, in this group of SAPs, the absorption capacity was the highest. On the other hand, although the formation of ester bonds in the SAPs containing CNF was higher than that containing BNC, the water absorption capacity in the first immersion time (i.e., 30 min) in the SAPs containing CNF was superior to that of BNC. From the abovementioned analysis, the amount of free hydrophilic groups in SAPs containing BNC was higher due to the higher crystallinity of these nanofibers and should enable a higher water absorption. However, the presence of BNC in the entrance of the holes, as shown by SEM analysis, caused the bacterial cellulose to strongly absorb water and quickly swell. As a consequence, the entrance of the holes could be blocked by swollen BNC. For this reason, in short-term absorption, even though the SAP containing BNC had more free hydrophilic groups, it showed less absorption capacity than that containing CNF.

However, over a longer period (i.e., 24 h), water was able to penetrate the pores, giving rise to a higher FSC than SAPs containing CNF. The studies conducted on hydrogels containing nanocellulose, especially CNF, showed that, due to hornification (namely, the phenomenon that the fibrils in nanocellulose are connected to each other due to hydrogen bonding and block the penetration path of water), the absorption capacity of hydrogels would sharply decrease [35,36]. To prevent this phenomenon, usually hydrogels are dried by a freeze-drying method [37]. However, in this research, the absorption capacity of the oven-dried SAPs was much higher than that of the freeze-dried SAPs. To analyze this finding, two important mechanisms could be involved: (i) the first mechanism is related to the fact that during the drying of hydrogels in the oven, numerous holes were created with almost-regular size and regular distribution in the SAPs, while the morphology of the freeze-dried SAPs was completely irregular, consistent with SEM images. These irregular holes, in terms of size and distribution, caused a little disturbance in the process of water absorption. Meanwhile, during freeze-drying, two important events could be occurring: a possible increase in the crystallinity of the manufactured SAPs and an increase in the number of hydrogen bonds between the hydroxyl groups of SA and nanocellulose [38]. There is also a possibility of inter-molecular hydrogen bonds in cellulose during lyophilization. During the last few years, several studies have been reported for hydrogels with a triple network. The mechanism is that the first network, which is mainly created by covalent bonding, plays the role of necessary strength and stiffness in the hydrogel [39], whereas the second network plays the role of flexibility in the mesh [39,40]. The third network, which has recently attracted the attention of researchers, is formed by freeze-drying and thawing the hydrogel *n* times [41,42,43]. As a result, the crystallinity of the hydrogel and the number of its hydrogen bonds increase simultaneously [38]. The purpose is to increase the mechanical properties of the hydrogel, but the water absorption capacity of the hydrogels made in this way is limited due to the presence of many hydrogen bonds and the blocking of hydroxyl groups, as well as the increase in the crystallinity of the hydrogels. (ii) Another possible mechanism is related to the fact that in all hydrogels subject to hornification, the nanofiber amount is very high (>10% by weight) [44]. However, in this research, hornification caused by oven-drying had a limited effect on the water absorption capacity compared to the increase in crystallinity and the increase in the number of hydrogen bonds caused by freeze-drying. Our results showed that in distilled water the FSC was greater than that in a saline solution. Studies have shown that the water absorption capacity of SAPs in saline solutions is much lower than their absorption capacity in distilled water [2,3,4,5]. When the SAPs are placed in electrolyte solutions or when different ions are present in water, the superabsorbent network is contracted, which interferes with the process of electrostatic anion–anion repulsion [45,46].

The results of CRC showed that this feature is relevant in the oven-dried SAPs containing CNC. Retention capacity refers to the ability to hold water. The water absorption process in SAPs can be classified as absorbed, semi-absorbed and free water [47]. The absorbed water depends on the number of hydrophilic groups, such as hydroxyl, carboxyl and amide, while the amount of free water has a direct relationship with the porosity percentage. Semi-absorbed water is a combination of absorbed and free water. Free water is easily removed by centrifugal force [47]. Therefore, two key factors affect the retention capacity of SAPs: porosity and the number of hydrophilic groups [47]. The porosity has an inverse relationship, while the number of hydrophilic groups has a direct relationship with the retention capacity [47]. Based on the results, the maximum and minimum GF percentage was in the freeze-dried SAPs containing CNF and the oven-dried SAPs containing CNC, respectively. GF is mainly affected by the cross-linker’s percentage under a direct relationship [48]; the greater the number of the cross-linkers, the greater the GF percentage. Studies have shown that the GF usually has an inverse relationship with the absorption capacity. Usually the GF in SAPs with a high absorption capacity is slightly lower than the GF in SAPs with a low absorption capacity [48]. In addition to the cross-linking agent, it is expected that any other factor that can affect the internal reactions of hydrogels can also affect the GF. In this research, the cross-linker percentage was constant, but CNF with the ability to form high hydrogen and ester bonds with SA caused the SAPs to have the highest GF percentage. In SAPs containing CNC, GF percentage was minimal since the mentioned reactions were minimal. As such, the dissolution of the SAP components in the samples containing CNF was very low due to the presence of many hydrogen and ester bonds, while the dissolution of the SAPs components in the samples containing CNC was higher due to the lack of those bonds. In fact, freeze-dried SAPs were more crystalline than the oven-dried counter parts, which can explain the increased GF in the former SAPs.

Based on the outcomes of AUL and salt sensitivity index, it could be expected that SAPs with a high absorption capacity would also have a high absorption capacity under load. Therefore, the highest AUL in the oven-dried SAPs and in SAPs containing CNC were in line with our expectations. As mentioned earlier, the lower the salt sensitivity index, the more sensitive the hydrogel is to sodium ions and *vice versa*. Thus, the highest salt sensitivity index in the freeze-dried SAPs made them less sensitive to the Na^+^ ions. This could be explained for these SAPs by a major role exerted by porosity in water absorbency compared to the hydrophilic groups. Indeed, during lyophilization, the crystallinity index is increased, which is inversely correlated with water absorbency [38]. However, in the oven-dried SAPs, the absorption process is a combination of real absorption (by hydrophilic groups) and water penetration into the pores. Therefore, the more the hydrophilic groups affect the water absorption process, the higher the sensitivity to salt.

The 24 h/30 min FSC ratio, in fact, confirmed the dissolving percentage. The lower this ratio is (≤100% or around 100%), the higher the dissolution rate. A number >100% indicates partial or non-dissolution of the hydrogel components in water. In distilled water, this ratio was slightly above 100% (i.e., 101% to 106%), while in a saline solution, it ranged from 120–130%. When the SAP powder was immersed in distilled water, the created anion–anion repulsion caused repulsion between the polymer chains [5]. In a saline solution, due to Na^+^ reducing repulsion and consequently polymer dissolution, the swelling percentage decreased and the separation of polymer chains from each other decreased. In this case, the hydrogel components remained in place to absorb water, which explains the high values of this ratio. Moreover, the 24 h/30 min FSC ratio in distilled water was lower in the oven-dried SAPs compared with the freeze-dried ones. This is ascribable to the intramolecular hydrogen bonds formed in SA and nanocellulose, as well as the increase in crystallinity of the freeze-dried SAPs. With increasing crystallinity and the increase in the number of intramolecular hydrogen bonds, the ability of water molecules to dissolve hydrogel components decreased, thus showing that this ratio in the absorption of distilled water depended on the degree of solubility of the hydrogel components and, in general, on the GF. However, since the solubility in saline solution was very small, this ratio in a saline solution was highly dependent on the absorption speed, as occurred in the oven-dried SAPs containing BNC. The SEM images showed that the entrance of the holes in this type of SAP was blocked by BNC, which, in early immersion times did not yet reach a maximum swelling, thus determining a low rate of absorption in the early period of immersion.

The lowest value of this ratio in a saline solution was found to be in the oven-dried SAPs containing CNF. This SAP had a high absorption rate and could be expected to show a 24 h/30 min FSC ratio low. However, since the dissolution rate in distilled water was also high, the absorption at 24 h was lower than that at 30 min and the 24 h/30 min FSC ratio was <100. The highest value of this ratio was found in the freeze-dried SAPs containing BNC, and the lowest in the oven-dried SAPs containing CNC. Considering the high reactivity of BNC, as well as the increase in crystallinity and the number of hydrogen bonds by freeze-drying, it can be hypothesized that this type of SAP will have a low solubility and, as a result, a higher 24 h/30 min FSC ratio. In addition, due to the lack of hydrogen and ester bonds related to the higher crystallinity of CNC, it was expected that the oven-dried SAPs containing CNC would have higher solubility and a lower 24 h/30 min FSC ratio. The CRC/FSC ratio was maximum in the oven-dried SAPs containing BNC and minimum in the freeze-dried SAPs containing BNC and CNF. The CRC/FSC ratio of the freeze-dried SAPs was higher than that of the oven-dried SAPs. The most important difference occurred in the retention capacity of the SAPs dried by these two methods, which caused the CRC/FSC ratio in the oven-dried SAPs to be higher. Even though the SAPs containing CNC had the highest retention capacity, the CRC/FSC ratio in this SAP was minimal. The CRC/FSC ratio in the SAPs containing BNC and CNF was higher than that containing CNC. The existence of many hydrogen and ester bonds between the hydroxyl groups of CNF with the hydroxyl and carboxyl groups of SA prevented the separation of water from the hydrogel, by the centrifugal force of the centrifuge. Moreover, the blocked holes in the SAPs containing BNC by the fibers concurred with the high CRC/FSC ratio in these SAPs and with the difficulty in the inlet/outlet of water. Although AUL was higher in the oven-dried SAPs than in the freeze-dried ones, the AUL/FSC ratio was higher in the freeze-dried SAPs than the oven-dried ones. This can be explained by the elastic properties of these types of SAPs, which moved the piston of the device during absorption under load, and from their rheological properties. In fact, the larger the distance between the piston of the device and the absorbent powder layer during the absorption process, the greater the absorption under load. As a secondary reason, freeze-dried SAPs had a large pore size, which affected capillarity in the first seconds of the process.

The results of the water absorption rate corroborated with an accordance to Fick’s law for these SAPs [10,11,12,13,14,15,16,17,18,19,20,21,22,23,24,25,26,27,28,29,30,31,32,33,34,35,36,37,38,39,40,41,42,43,44,45,46,47,48,49]. The water diffusion coefficient in the SAPs containing CNF and BNC was higher than that containing CNC, which indicated the higher velocity of water absorption in this type of SAP. Although SAPs containing CNF and BNC had absorbed less water, they absorbed water at a faster rate compared to SAPs containing CNC. This is explained by the larger size of the holes in SAPs containing CNF and BNC compared to CNC. Another point is that although the entrance of the holes of the SAPs containing BNC was blocked by these nanofibers, the larger size of the holes had a better effect on the rate of water absorption than blocking the holes. The water diffusion coefficient in the freeze-dried SAPs was higher than that of the oven-dried SAPs, indicating the higher rate of water absorption in this type of SAP. This can be related to the lightness and large size of the holes, which caused a very large amount of water to be absorbed in a short period of time by these types of SAPs.

The results of the rheological property investigation showed that the loss modulus was much lower than the storage modulus. This behavior in SAPs is usually shown by strong gels, and researchers have reported that the loss moduli are higher than the storage moduli in low-viscosity and watery gels [44,45,46,47,48,49,50]. More than any other factors, the storage modulus and loss modulus are directly related to the absorption capacity of the SAPs [50], and indirectly related to factors, such as the number and size of the holes, the porosity percentage and the type and quantity of reactions, among others. Therefore, based on the results, the higher storage modulus of the SAPs containing BNC can be related to the lower water absorption capacity, as a lower absorption capacity means a stronger gel, and to the high aspect ratio of these fibers [31]. The good viscoelastic properties of BNC concur with the higher storage modulus, which represents the elastic properties of the material [31]. SAPs containing BNC had a uniform distribution of the holes, which affected the viscoelastic properties of these SAPs. However, although the absorption capacity of the SAPs containing CNC was higher than that of the SAPs containing CNF, it is likely that the extraordinary elastic properties of CNC had a much greater effect on their storage modulus compared to the absorption capacity. Even if there is a slight difference between the loss modulus of SAPs containing various types of nanocellulose, the higher loss modulus of SAPs containing CNC compared to the rest of the SAPs can be attributed to the lower CRC/FSC ratio, meaning that the gel resistance after swelling is lower and there is probably more water in the network structure of the hydrogel [50]. Researchers have presented a similar report regarding the high loss modulus in SAPs with low resistance [49,50]. Overall, the CRC/FSC ratio in all these SAPs was high (≥69%), and the presented analyses were only for the comparison of SAPs containing various types of nanocellulose. The results also showed that freeze-dried SAPs had a better storage and loss modulus than oven-dried SAPs. It has been reported that freeze-drying increases the crystallinity of the system, which could be related to improved elastic properties [38]. However, the freeze-dried SAPs had a lower water absorption capacity than the oven-dried SAPs, and this is the main reason for their higher storage modulus. On the other hand, the CRC/FSC ratio in the freeze-dried SAPs was lower than that found in the oven-dried SAPs; therefore, the swollen gel in this state is close to the watery state. Researchers have in fact reported a high loss modulus in low-viscosity SAPs [49,50].

In recent years, the attention of researchers has been increasingly focused on more sustainable routes to improve the performance of bio-based polymers, by means of a variegated set of strategies, such as blending/compositing [51], up to replacing current formulations and products with greener ones [52,53], also in the use of cellulose and nanocellulose for biomedical products and SAPs [53,54]. The interplay of cellulosic molecules in SAPs is very complex, but it retains a high potential to develop eco-friendly SAPs and deserves further investigation. Finally, other eco-compatible methods to control hydrogel porosity and thus improve water absorption could be added, such as ice templating [55], which would possibly enhance the performance of these SAPs.

## 5. Conclusions

In this study, the effects of different types of nanocellulose (CNC, CNF and BNC) along with two different drying methods (i.e., oven-drying and freeze-drying) on the properties of AA/SA–based SAPs were investigated. The results of FTIR spectroscopy confirmed the graft-copolymerization between AA and SA. The SEM results showed a micro-porous structure in the SAPs. The size of the holes in the SAPs made with CNC was much smaller than those containing CNF and BNC. The results of absorption behavior also highlighted that the oven-dried SAPs had better properties compared to the freeze-dried ones. Overall, SAPs containing CNC had better absorption properties than the other SAPs. The freeze-dried SAPs had better rheological properties in comparison to the oven-dried ones. SAPs containing BNC and CNC had a maximum storage modulus and loss modulus, respectively. In general, the oven-dried SAPs containing CNC had very good practical properties. In hygienic products, such as baby diapers, sanitary napkin and adult incontinence pads these SAPs could be considered as alternatives to replace commercial powders. This will reduce the environmental problems caused by commercial powders that are made of 100% oil-based materials.

## Figures and Tables

**Table 1:** The characteristics of different types of nanocellulose.

Nanocellulose Type	Length (μm)	Diameter (nm)	Aspect Ratio (nm/nm)	Crystallinity (%)
CNC	0.3	5	60	85
CNF	~2.0	15	~133	65
BNC	~3.0	10	~300	75

**Table 2:** Summarized results (average) for super absorption polymers (SAPs) combined with diverse nanocellulose sources, i.e., cellulose nanocrystal (CNC), cellulose nanofiber (CNF) and bacterial nanocellulose (BNC) of all absorption properties investigated: Free swelling capacity (FSC), Centrifuge retention capacity (CRC), Gel fraction (GF), Absorption under load (AUL), Salt sensitivity index (SSI), of differently dried samples with different aqueous media: Oven-drying (O-D), Freeze-drying (F-D), saline solution (SS), distilled water (dH_2_O).

Tests	CNC+ O-D	CNC+ F-D	CNF+ O-D	CNF+ F-D	BNC+ O-D	BNC+ F-D
FSC in SS, 30 min (g/g)	98.87	54.25	87.34	44.93	77.05	45.61
FSC in SS, 24 h (g/g)	127.55	68.15	98.57	57.27	106.97	55.77
FSC in dH_2_O, 30 min (g/g)	742.82	243.67	572.87	209.4	553.55	205.6
FSC in dH_2_O, 24 h (g/g)	690.42	265.57	583.72	216.1	529.42	240.35
CRC (g/g)	73.22	38.94	67.01	31.58	60.29	31.62
GF (%)	68.33	73.33	73.33	81.66	73.33	76.66
AUL (g/g)	20.12	13.53	17.75	12.73	15.36	10.93
SSI (%)	13.31	22.31	15.24	21.46	13.91	22.18
FSC ratio|_24 h/30 min_ in SS (%)	129.24	125.6	112.9	127.6	139.12	122.28
FSC ratio|_24 h/30 min_ in dH_2_O (%)	92.95	109.18	101.88	103.3	95.64	116.96
CRC/FSC (%)	74.19	71.80	76.70	70.43	78.39	69.32
AUL/FSC (%)	20.31	24.97	20.31	28.51	19.96	23.94

## Data Availability

The data presented in this study are available on request from the corresponding authors.
